# *catena*-Poly[[tetra­kis­(hexa­methyl­phospho­ramide-κ*O*)bis­(nitrato-κ^2^ *O*,*O*′)cerium(III)] [silver(I)-di-μ-sulfido-tungstate(VI)-di-μ-sulfido]]

**DOI:** 10.1107/S1600536810044260

**Published:** 2010-11-27

**Authors:** Hongyang Wei, Jinfang Zhang, Chi Zhang

**Affiliations:** a Institute of Molecular Engineering and Advanced Materials, School of Chemical Engineering, Nanjing University of Science and Technology, 200 Xiaolingwei, Nanjing 210094, Jiangsu, People’s Republic of China; b Institute of Science and Technology, Jiangsu University, 301 Xuefu Road, Zhenjiang 212013, People’s Republic of China

## Abstract

Hexa­methyl­phospho­ramide (hmp), tetra­thio­tungstate, silver sulfide and cerium nitrate were self-assembled to form a one-dimensional anionic [Ag_4_W_4_S_16_]_*n*_^4*n*−^ chain in the title compound, {[Ce(NO_3_)_2_(C_6_H_18_N_3_OP)_4_][AgWS_4_]}_*n*_. The asymmetric unit contains four crystallographically independent [Ce(hmp)_4_(NO_3_)_2_]^+^ cations, which leads to a one-dimensional polymeric anionic chain having a tetra­valent [W_4_S_16_Ag_4_] repeat unit. Each central Ce atom is coordinated by eight O atoms from two chelating nitrate and four hmp ligands, which gives rise to a distorted square-anti­prismatic structure. The polymeric chain with average W—Ag—W and Ag—W—Ag bond angles of 163.75 and 151.84°, respectively, presents a distorted linear configuration. The title complex with a non-centrosymmetric structure, is analogous to the Yb, Y, Eu, Nd, La and Dy isomorphs, which exhibit centrosymmetric structures.

## Related literature

For one-dimensional Mo(W)/S/Ag anionic polymers, see: Niu *et al.* (2004 ▶). For their unique properties, see: Zhang, Song *et al.* (2007 ▶). For analogous centrosymmetric complexes, see: Cao *et al.* (2007 ▶); Zhang, Cao *et al.* (2007 ▶); Zhang, Qian *et al.* (2007 ▶); Tang, Zhang & Zhang (2008 ▶); Tang, Zhang, Zhang & Lu (2008 ▶); Wei *et al.* (2010 ▶).
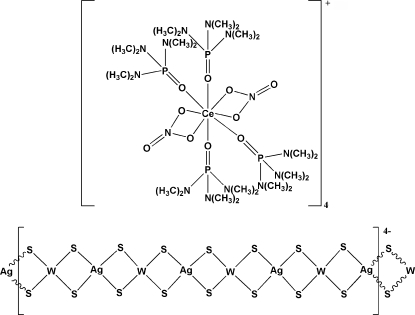


## Experimental

### 

#### Crystal data

[Ce(NO_3_)_2_(C_6_H_18_N_3_OP)_4_][AgWS_4_]
*M* *_r_* = 1400.92
Monoclinic, 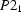

*a* = 15.639 (3) Å
*b* = 30.002 (6) Å
*c* = 22.532 (4) Å
β = 90.53 (3)°
*V* = 10572 (3) Å^3^
*Z* = 8
Mo *K*α radiation
μ = 3.72 mm^−1^
*T* = 153 K
0.30 × 0.22 × 0.20 mm

#### Data collection

Rigaku Saturn724+ diffractometer
Absorption correction: multi-scan (*CrystalClear*; Rigaku, 2007 ▶) *T*_min_ = 0.390, *T*_max_ = 0.475
49779 measured reflections
34142 independent reflections
32497 reflections with *I* > 2σ(*I*)
*R*_int_ = 0.024

#### Refinement

*R*[*F* ^2^ > 2σ(*F* ^2^)] = 0.034
*wR*(*F* ^2^) = 0.068
*S* = 1.06
34142 reflections
2221 parameters
1 restraint
H-atom parameters constrained
Δρ_max_ = 0.74 e Å^−3^
Δρ_min_ = −0.96 e Å^−3^
Absolute structure: Flack (1983 ▶), 14534 Friedel pairs
Flack parameter: 0.286 (4)

### 

Data collection: *CrystalClear* (Rigaku, 2007 ▶); cell refinement: *CrystalClear*; data reduction: *CrystalClear*; program(s) used to solve structure: *SHELXTL* (Sheldrick, 2008 ▶); program(s) used to refine structure: *SHELXTL*; molecular graphics: *SHELXTL*; software used to prepare material for publication: *SHELXTL*.

## Supplementary Material

Crystal structure: contains datablocks I, global. DOI: 10.1107/S1600536810044260/zq2066sup1.cif

Structure factors: contains datablocks I. DOI: 10.1107/S1600536810044260/zq2066Isup2.hkl

Additional supplementary materials:  crystallographic information; 3D view; checkCIF report

## Figures and Tables

**Fig. 1.**
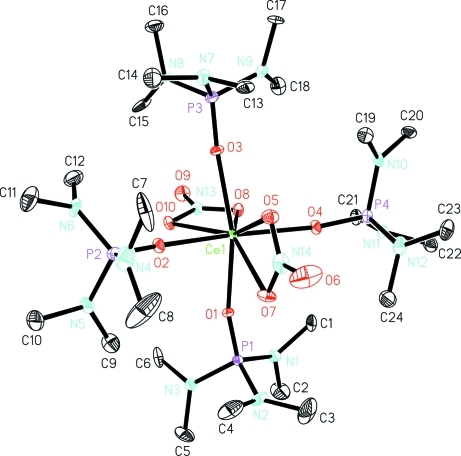
The molecular structure of one [Ce(hmp)4(NO3)2]+ cation in the title compound, with atom labels and 30% probability displacement ellipsoids. All H atoms have been omitted.

**Fig. 2.**
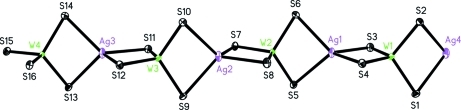
The molecular structure of a portion of the anionic chain in the title compound, with atom labels and 30% probability displacement ellipsoids.

## supplementary crystallographic information

### Comment

One-dimensional Mo(W)/S/Ag anionic polymers have attracted much attention for their configurational isomerism (Niu *et al.*, 2004) and unique properties as functional materials, such as third-order nonlinear optical (NLO) materials (Zhang, Song & Wang, 2007). Different solvent-coordinated rare-earth cations proved effective to obtain various configurations of anionic chains (Niu *et al.*, 2004). The title compound {4n[Ce(hmp)_4_(NO_3_)_2_][Ag_4_W_4_S_16_]_n_} (hmp = hexamethylphosphoramide) with a wave-like anionic chain was prepared by following such route using Ce(III)-hmp complex as counterion.

Each Ce^3-^ in the cation is coordinated by eight O atoms from two nitrate and four hmp ligands (Fig. 1). Parts of dimethylamine groups from hmp ligands have large librations reflecting a small degree of thermal disorder. The cation in the title compound contains four univalent [Ce(hmp)_4_(NO_3_)_2_]^+^, which leads to a 1-D anionic chain having a tetravalent repeat unit. As illustrated in Fig. 2, the anionic chain in the title compound has a distorted linear configuration with average W—Ag—W and Ag—W—Ag angles of 163.75 and 151.84 °, respectively. The title complex is analogous to Yb (Cao *et al.* 2007), Y (Zhang, Cao *et al.* 2007), Eu (Zhang, Qian *et al.* 2007), Nd (Tang, Zhang & Zhang, 2008), La (Tang, Zhang, Zhang & Lu, 2008) and Dy (Wei *et al.* 2010) isomorphs.

### Experimental

1 mmol Ag_2_S was added to a solution of [NH_4_]_2_WS_4_ (2 mmol in 30 mL hmp) with thorough stir for 6 h. The solution underwent an additional stir for two minute after 1 mmol Ce(NO_3_)_3_.6H_2_O was added. After filtration the orange-red filtrate was carefully laid on the surface with 30 ml *i*-PrOH. orange block crystals were obtained after ten days.

### Refinement

H atoms were positioned geometrically and refined with riding model, with *U*_iso_ = 1.5*U*_eq_ for methyl H atoms and 0.96 Å for C—H bonds.

### Crystal data

**Table:**

[Ce(NO_3_)_2_(C_6_H_18_N_3_OP)_4_][AgWS_4_]	*F*(000) = 5576
*M**_r_* = 1400.92	*D*_x_ = 1.760 Mg m^−^^3^
Monoclinic, *P*2_1_	Mo *K*α radiation, λ = 0.71073 Å
*a* = 15.639 (3) Å	Cell parameters from 45199 reflections
*b* = 30.002 (6) Å	θ = 2.8–29.0°
*c* = 22.532 (4) Å	µ = 3.72 mm^−^^1^
β = 90.53 (3)°	*T* = 153 K
*V* = 10572 (3) Å^3^	Block, orange
*Z* = 8	0.3 × 0.22 × 0.20 mm

### Data collection

**Table:**

Rigaku Saturn724+ (2x2 bin mode) diffractometer	34142 independent reflections
Radiation source: fine-focus sealed tube	32497 reflections with *I* > 2σ(*I*)
graphite	*R*_int_ = 0.024
ω scans	θ_max_ = 25.4°, θ_min_ = 2.8°
Absorption correction: multi-scan (*CrystalClear*; Rigaku, 2007)	*h* = −12→18
*T*_min_ = 0.390, *T*_max_ = 0.475	*k* = −35→36
49779 measured reflections	*l* = −27→25

### Refinement

**Table:**

Refinement on *F*^2^	Secondary atom site location: difference Fourier map
Least-squares matrix: full	Hydrogen site location: inferred from neighbouring sites
*R*[*F*^2^ > 2σ(*F*^2^)] = 0.034	H-atom parameters constrained
*wR*(*F*^2^) = 0.068	*w* = 1/[σ^2^(*F*_o_^2^) + (0.0224*P*)^2^ + 4.7571*P*] where *P* = (*F*_o_^2^ + 2*F*_c_^2^)/3
*S* = 1.06	(Δ/σ)_max_ = 0.003
34142 reflections	Δρ_max_ = 0.74 e Å^−^^3^
2221 parameters	Δρ_min_ = −0.96 e Å^−^^3^
1 restraint	Absolute structure: Flack (1983), 14534 Friedel pairs
Primary atom site location: structure-invariant direct methods	Flack parameter: 0.286 (4)

### Special details

**Table:**

Geometry. All e.s.d.'s (except the e.s.d. in the dihedral angle between two l.s. planes) are estimated using the full covariance matrix. The cell e.s.d.'s are taken into account individually in the estimation of e.s.d.'s in distances, angles and torsion angles; correlations between e.s.d.'s in cell parameters are only used when they are defined by crystal symmetry. An approximate (isotropic) treatment of cell e.s.d.'s is used for estimating e.s.d.'s involving l.s. planes.
Refinement. Refinement of *F*^2^ against ALL reflections. The weighted *R*-factor *wR* and goodness of fit *S* are based on *F*^2^, conventional *R*-factors *R* are based on *F*, with *F* set to zero for negative *F*^2^. The threshold expression of *F*^2^ > σ(*F*^2^) is used only for calculating *R*-factors(gt) *etc*. and is not relevant to the choice of reflections for refinement. *R*-factors based on *F*^2^ are statistically about twice as large as those based on *F*, and *R*- factors based on ALL data will be even larger.

### Fractional atomic coordinates and isotropic or equivalent isotropic displacement parameters (Å^2^)

**Table:**

	*x*	*y*	*z*	*U*_iso_*/*U*_eq_	
W1	0.70667 (2)	0.670558 (10)	0.635629 (14)	0.01653 (8)	
W2	0.70710 (2)	0.718307 (10)	0.384943 (14)	0.01684 (8)	
W3	0.71320 (2)	0.680506 (10)	0.132285 (14)	0.01660 (8)	
W4	0.71030 (2)	0.726819 (9)	−0.117449 (14)	0.01602 (8)	
Ag1	0.70624 (5)	0.70662 (2)	0.51475 (3)	0.02993 (18)	
Ag2	0.71221 (5)	0.68628 (2)	0.26209 (3)	0.03115 (18)	
Ag3	0.71502 (5)	0.71867 (2)	0.01258 (3)	0.03065 (18)	
Ag4	0.70672 (5)	0.68569 (2)	0.76465 (3)	0.03160 (18)	
S1	0.59159 (15)	0.65587 (8)	0.68776 (10)	0.0265 (5)	
S2	0.82175 (15)	0.65644 (8)	0.68948 (10)	0.0244 (5)	
S3	0.70493 (16)	0.74260 (7)	0.61376 (10)	0.0240 (5)	
S4	0.70920 (15)	0.62697 (7)	0.55689 (10)	0.0233 (5)	
S5	0.59056 (15)	0.73146 (9)	0.43625 (10)	0.0304 (6)	
S6	0.82233 (15)	0.73143 (8)	0.43943 (10)	0.0262 (5)	
S7	0.70824 (16)	0.76450 (7)	0.30862 (9)	0.0237 (5)	
S8	0.70711 (17)	0.64732 (7)	0.35904 (10)	0.0279 (5)	
S9	0.59795 (15)	0.66429 (8)	0.18283 (10)	0.0260 (5)	
S10	0.82968 (15)	0.66679 (7)	0.18527 (10)	0.0236 (5)	
S11	0.70992 (15)	0.75284 (7)	0.11250 (10)	0.0214 (5)	
S12	0.71504 (15)	0.63835 (7)	0.05201 (9)	0.0230 (5)	
S13	0.59544 (15)	0.74205 (7)	−0.06550 (10)	0.0236 (5)	
S14	0.82577 (15)	0.74540 (8)	−0.06615 (10)	0.0266 (5)	
S15	0.70532 (15)	0.76722 (7)	−0.19945 (10)	0.0243 (5)	
S16	0.71292 (15)	0.65425 (7)	−0.13326 (10)	0.0230 (5)	
Ce1	0.23943 (3)	0.530118 (13)	0.432688 (18)	0.01342 (10)	
P1	0.17428 (13)	0.43188 (7)	0.53337 (9)	0.0197 (4)	
P2	0.47960 (13)	0.52705 (7)	0.47157 (9)	0.0268 (5)	
P3	0.30186 (15)	0.60588 (7)	0.30129 (10)	0.0229 (5)	
P4	0.01650 (14)	0.58453 (7)	0.44156 (9)	0.0181 (5)	
O1	0.2087 (3)	0.46413 (17)	0.4896 (2)	0.0179 (12)	
O2	0.3877 (3)	0.52404 (18)	0.4519 (2)	0.0242 (13)	
O3	0.2779 (4)	0.57219 (17)	0.3469 (2)	0.0203 (12)	
O4	0.0975 (3)	0.55851 (17)	0.4391 (2)	0.0208 (13)	
O5	0.2604 (4)	0.6089 (2)	0.4817 (3)	0.0306 (15)	
O6	0.2435 (6)	0.6213 (3)	0.5756 (3)	0.071 (3)	
O7	0.2374 (4)	0.55348 (19)	0.5427 (3)	0.0300 (15)	
O8	0.1566 (4)	0.48881 (18)	0.3496 (2)	0.0229 (13)	
O9	0.2122 (4)	0.44519 (19)	0.2840 (3)	0.0328 (16)	
O10	0.2868 (4)	0.46843 (18)	0.3593 (2)	0.0212 (13)	
N1	0.0763 (4)	0.4198 (2)	0.5144 (3)	0.0273 (16)	
N2	0.1759 (5)	0.4496 (2)	0.6025 (3)	0.0328 (17)	
N3	0.2337 (4)	0.3872 (2)	0.5358 (3)	0.0232 (15)	
N4	0.5043 (5)	0.5670 (3)	0.5147 (3)	0.051 (2)	
N5	0.5035 (5)	0.4798 (3)	0.5038 (3)	0.054 (2)	
N6	0.5411 (4)	0.5344 (2)	0.4153 (3)	0.0337 (16)	
N7	0.3637 (5)	0.6472 (2)	0.3233 (3)	0.0259 (17)	
N8	0.3554 (5)	0.5809 (2)	0.2485 (3)	0.0362 (19)	
N9	0.2145 (5)	0.6306 (2)	0.2772 (3)	0.0238 (16)	
N10	0.0143 (4)	0.6252 (2)	0.3931 (3)	0.0197 (15)	
N11	−0.0633 (4)	0.5508 (2)	0.4284 (3)	0.0203 (15)	
N12	0.0054 (5)	0.6066 (2)	0.5074 (3)	0.0285 (18)	
N13	0.2168 (5)	0.4668 (2)	0.3295 (3)	0.0203 (16)	
N14	0.2473 (6)	0.5946 (3)	0.5344 (4)	0.039 (2)	
C1	0.0184 (5)	0.4534 (3)	0.4900 (4)	0.033 (2)	
H1A	−0.0202	0.4630	0.5203	0.049*	
H1B	−0.0136	0.4408	0.4575	0.049*	
H1C	0.0509	0.4784	0.4762	0.049*	
C2	0.0327 (6)	0.3797 (3)	0.5369 (5)	0.059 (3)	
H2A	−0.0095	0.3700	0.5085	0.089*	
H2B	0.0053	0.3866	0.5738	0.089*	
H2C	0.0738	0.3564	0.5432	0.089*	
C3	0.1047 (8)	0.4672 (5)	0.6322 (4)	0.105 (6)	
H3A	0.0980	0.4981	0.6220	0.158*	
H3B	0.1133	0.4645	0.6743	0.158*	
H3C	0.0542	0.4511	0.6206	0.158*	
C4	0.2566 (7)	0.4626 (4)	0.6307 (4)	0.057 (3)	
H4A	0.2553	0.4551	0.6721	0.085*	
H4B	0.2648	0.4941	0.6263	0.085*	
H4C	0.3029	0.4470	0.6121	0.085*	
C5	0.2358 (6)	0.3552 (3)	0.5853 (4)	0.043 (2)	
H5A	0.2938	0.3462	0.5929	0.065*	
H5B	0.2020	0.3296	0.5751	0.065*	
H5C	0.2130	0.3690	0.6202	0.065*	
C6	0.2675 (5)	0.3682 (3)	0.4811 (4)	0.033 (2)	
H6A	0.2370	0.3412	0.4717	0.049*	
H6B	0.3271	0.3615	0.4865	0.049*	
H6C	0.2605	0.3891	0.4493	0.049*	
C7	0.4995 (7)	0.6124 (4)	0.4926 (6)	0.071 (4)	
H7A	0.5337	0.6315	0.5175	0.106*	
H7B	0.4412	0.6223	0.4930	0.106*	
H7C	0.5206	0.6134	0.4528	0.106*	
C8	0.4866 (8)	0.5612 (5)	0.5782 (5)	0.118 (6)	
H8A	0.4314	0.5733	0.5870	0.177*	
H8B	0.5295	0.5764	0.6013	0.177*	
H8C	0.4875	0.5300	0.5878	0.177*	
C9	0.4446 (6)	0.4508 (3)	0.5306 (4)	0.039 (2)	
H9A	0.4422	0.4570	0.5723	0.059*	
H9B	0.4624	0.4206	0.5246	0.059*	
H9C	0.3891	0.4552	0.5130	0.059*	
C10	0.5930 (6)	0.4686 (4)	0.5180 (5)	0.091 (5)	
H10A	0.6010	0.4370	0.5147	0.137*	
H10B	0.6064	0.4780	0.5577	0.137*	
H10C	0.6301	0.4836	0.4907	0.137*	
C11	0.6314 (6)	0.5454 (3)	0.4192 (5)	0.058 (3)	
H11A	0.6464	0.5645	0.3867	0.087*	
H11B	0.6646	0.5185	0.4174	0.087*	
H11C	0.6429	0.5604	0.4560	0.087*	
C12	0.5168 (6)	0.5193 (3)	0.3558 (3)	0.047 (2)	
H12A	0.5483	0.4929	0.3462	0.070*	
H12B	0.5294	0.5423	0.3276	0.070*	
H12C	0.4566	0.5130	0.3547	0.070*	
C13	0.3310 (6)	0.6770 (3)	0.3683 (4)	0.028 (2)	
H13A	0.3592	0.7053	0.3654	0.042*	
H13B	0.3416	0.6644	0.4069	0.042*	
H13C	0.2706	0.6809	0.3625	0.042*	
C14	0.4561 (5)	0.6414 (3)	0.3275 (4)	0.045 (2)	
H14A	0.4694	0.6202	0.3582	0.068*	
H14B	0.4824	0.6695	0.3368	0.068*	
H14C	0.4773	0.6307	0.2903	0.068*	
C15	0.3602 (6)	0.5341 (2)	0.2407 (3)	0.040 (2)	
H15A	0.4189	0.5254	0.2361	0.060*	
H15B	0.3281	0.5257	0.2060	0.060*	
H15C	0.3369	0.5194	0.2748	0.060*	
C16	0.3807 (7)	0.6064 (3)	0.1961 (4)	0.053 (3)	
H16A	0.3372	0.6038	0.1659	0.079*	
H16B	0.4337	0.5949	0.1813	0.079*	
H16C	0.3879	0.6372	0.2067	0.079*	
C17	0.2135 (6)	0.6723 (3)	0.2440 (4)	0.030 (2)	
H17A	0.1635	0.6891	0.2541	0.045*	
H17B	0.2125	0.6659	0.2022	0.045*	
H17C	0.2637	0.6892	0.2537	0.045*	
C18	0.1435 (6)	0.6006 (3)	0.2603 (4)	0.040 (2)	
H18A	0.1503	0.5913	0.2198	0.059*	
H18B	0.0902	0.6161	0.2642	0.059*	
H18C	0.1440	0.5750	0.2858	0.059*	
C19	0.0815 (6)	0.6584 (3)	0.3958 (4)	0.028 (2)	
H19A	0.0605	0.6847	0.4151	0.042*	
H19B	0.0990	0.6658	0.3563	0.042*	
H19C	0.1294	0.6467	0.4178	0.042*	
C20	−0.0589 (6)	0.6357 (3)	0.3549 (4)	0.028 (2)	
H20A	−0.0394	0.6427	0.3158	0.043*	
H20B	−0.0889	0.6609	0.3709	0.043*	
H20C	−0.0967	0.6105	0.3531	0.043*	
C21	−0.0621 (5)	0.5211 (3)	0.3774 (3)	0.0266 (19)	
H21A	−0.0906	0.5351	0.3444	0.040*	
H21B	−0.0908	0.4938	0.3871	0.040*	
H21C	−0.0039	0.5147	0.3670	0.040*	
C22	−0.1502 (6)	0.5590 (3)	0.4516 (4)	0.036 (2)	
H22A	−0.1761	0.5312	0.4622	0.054*	
H22B	−0.1843	0.5734	0.4216	0.054*	
H22C	−0.1464	0.5779	0.4860	0.054*	
C23	−0.0389 (7)	0.6479 (3)	0.5182 (4)	0.037 (2)	
H23A	−0.0016	0.6681	0.5390	0.055*	
H23B	−0.0886	0.6423	0.5417	0.055*	
H23C	−0.0561	0.6609	0.4810	0.055*	
C24	0.0269 (6)	0.5802 (3)	0.5609 (4)	0.032 (2)	
H24A	−0.0248	0.5707	0.5796	0.048*	
H24B	0.0595	0.5983	0.5880	0.048*	
H24C	0.0599	0.5547	0.5497	0.048*	
Ce2	0.24257 (3)	0.535742 (13)	0.950288 (18)	0.01308 (10)	
P5	0.20715 (14)	0.42160 (7)	1.02711 (9)	0.0202 (4)	
P6	0.47539 (12)	0.54651 (6)	0.99636 (8)	0.0175 (4)	
P7	0.29820 (14)	0.60994 (7)	0.81644 (9)	0.0194 (5)	
P8	0.01721 (14)	0.58537 (7)	0.96084 (10)	0.0189 (5)	
O11	0.2231 (3)	0.46756 (17)	1.0052 (2)	0.0174 (12)	
O12	0.3910 (3)	0.53180 (18)	0.9713 (2)	0.0202 (12)	
O13	0.2741 (4)	0.58384 (17)	0.8699 (2)	0.0206 (13)	
O14	0.0981 (3)	0.55967 (17)	0.9588 (2)	0.0171 (12)	
O15	0.1644 (4)	0.49599 (19)	0.8630 (2)	0.0217 (13)	
O16	0.2289 (5)	0.4617 (2)	0.7912 (3)	0.0419 (18)	
O17	0.2996 (4)	0.48047 (19)	0.8704 (3)	0.0263 (14)	
O18	0.2526 (4)	0.61465 (19)	0.9986 (2)	0.0222 (13)	
O19	0.2197 (5)	0.6304 (2)	1.0894 (3)	0.0455 (19)	
O20	0.2350 (4)	0.56068 (19)	1.0601 (2)	0.0253 (14)	
N15	0.1153 (4)	0.4051 (2)	0.9990 (3)	0.0306 (16)	
N16	0.2127 (4)	0.4222 (2)	1.1000 (3)	0.0289 (16)	
N17	0.2739 (4)	0.3827 (2)	1.0084 (3)	0.0280 (16)	
N18	0.4782 (4)	0.53218 (19)	1.0668 (3)	0.0236 (14)	
N19	0.5490 (4)	0.5231 (2)	0.9568 (3)	0.0342 (17)	
N20	0.4982 (5)	0.5995 (2)	0.9978 (3)	0.0222 (15)	
N21	0.3564 (5)	0.5802 (2)	0.7727 (3)	0.0318 (18)	
N22	0.3521 (5)	0.6546 (2)	0.8353 (3)	0.0258 (17)	
N23	0.2106 (5)	0.6241 (2)	0.7809 (3)	0.0306 (18)	
N24	0.0151 (4)	0.6245 (2)	0.9105 (3)	0.0226 (16)	
N25	−0.0618 (4)	0.5506 (2)	0.9497 (3)	0.0275 (17)	
N26	0.0065 (5)	0.6085 (2)	1.0263 (3)	0.0234 (16)	
N27	0.2304 (5)	0.4792 (2)	0.8397 (3)	0.0286 (18)	
N28	0.2359 (5)	0.6021 (3)	1.0508 (3)	0.0317 (19)	
C25	0.0494 (5)	0.4383 (3)	0.9877 (4)	0.036 (2)	
H25A	0.0289	0.4497	1.0248	0.054*	
H25B	0.0029	0.4249	0.9660	0.054*	
H25C	0.0729	0.4623	0.9648	0.054*	
C26	0.0806 (6)	0.3608 (3)	1.0119 (5)	0.059 (3)	
H26A	0.0462	0.3509	0.9790	0.089*	
H26B	0.0461	0.3623	1.0469	0.089*	
H26C	0.1268	0.3402	1.0183	0.089*	
C27	0.1935 (6)	0.3819 (3)	1.1343 (4)	0.043 (2)	
H27A	0.1333	0.3807	1.1421	0.065*	
H27B	0.2247	0.3826	1.1711	0.065*	
H27C	0.2100	0.3561	1.1120	0.065*	
C28	0.1960 (7)	0.4630 (3)	1.1334 (4)	0.041 (2)	
H28A	0.2343	0.4646	1.1668	0.062*	
H28B	0.1380	0.4628	1.1470	0.062*	
H28C	0.2047	0.4883	1.1082	0.062*	
C29	0.3532 (6)	0.3732 (3)	1.0395 (4)	0.044 (2)	
H29A	0.3994	0.3881	1.0200	0.066*	
H29B	0.3633	0.3416	1.0393	0.066*	
H29C	0.3494	0.3835	1.0797	0.066*	
C30	0.2786 (7)	0.3677 (4)	0.9459 (4)	0.068 (3)	
H30A	0.2992	0.3376	0.9446	0.102*	
H30B	0.3169	0.3868	0.9245	0.102*	
H30C	0.2227	0.3691	0.9280	0.102*	
C31	0.4357 (5)	0.4912 (3)	1.0834 (3)	0.033 (2)	
H31A	0.4236	0.4918	1.1251	0.050*	
H31B	0.4721	0.4663	1.0748	0.050*	
H31C	0.3832	0.4884	1.0613	0.050*	
C32	0.5563 (5)	0.5394 (3)	1.1024 (3)	0.038 (2)	
H32A	0.5993	0.5184	1.0906	0.057*	
H32B	0.5437	0.5352	1.1437	0.057*	
H32C	0.5767	0.5691	1.0963	0.057*	
C33	0.5327 (5)	0.4895 (3)	0.9126 (4)	0.042 (2)	
H33A	0.5593	0.4620	0.9245	0.062*	
H33B	0.5557	0.4989	0.8753	0.062*	
H33C	0.4721	0.4851	0.9084	0.062*	
C34	0.6399 (5)	0.5338 (3)	0.9642 (4)	0.050 (3)	
H34A	0.6644	0.5397	0.9260	0.075*	
H34B	0.6689	0.5090	0.9823	0.075*	
H34C	0.6460	0.5596	0.9890	0.075*	
C35	0.4626 (5)	0.6289 (2)	1.0424 (4)	0.032 (2)	
H35A	0.4107	0.6420	1.0274	0.049*	
H35B	0.5030	0.6520	1.0516	0.049*	
H35C	0.4506	0.6121	1.0776	0.049*	
C36	0.5230 (6)	0.6220 (3)	0.9422 (4)	0.043 (3)	
H36A	0.5594	0.6469	0.9514	0.065*	
H36B	0.4726	0.6324	0.9218	0.065*	
H36C	0.5530	0.6014	0.9174	0.065*	
C37	0.4301 (6)	0.5572 (3)	0.7973 (4)	0.053 (3)	
H37A	0.4815	0.5718	0.7847	0.079*	
H37B	0.4303	0.5269	0.7838	0.079*	
H37C	0.4275	0.5577	0.8399	0.079*	
C38	0.3534 (8)	0.5825 (4)	0.7079 (4)	0.064 (3)	
H38A	0.3500	0.5529	0.6918	0.095*	
H38B	0.4042	0.5968	0.6937	0.095*	
H38C	0.3041	0.5993	0.6955	0.095*	
C39	0.4142 (6)	0.6760 (3)	0.7968 (4)	0.044 (3)	
H39A	0.4643	0.6836	0.8195	0.066*	
H39B	0.3897	0.7025	0.7800	0.066*	
H39C	0.4295	0.6559	0.7655	0.066*	
C40	0.3224 (6)	0.6819 (3)	0.8827 (4)	0.037 (2)	
H40A	0.2920	0.7070	0.8667	0.055*	
H40B	0.3703	0.6921	0.9058	0.055*	
H40C	0.2850	0.6648	0.9074	0.055*	
C41	0.1994 (6)	0.6669 (3)	0.7506 (4)	0.038 (2)	
H41A	0.1473	0.6806	0.7635	0.057*	
H41B	0.1967	0.6621	0.7085	0.057*	
H41C	0.2468	0.6860	0.7599	0.057*	
C42	0.1428 (7)	0.5918 (3)	0.7673 (5)	0.050 (3)	
H42A	0.1355	0.5896	0.7250	0.075*	
H42B	0.0903	0.6015	0.7848	0.075*	
H42C	0.1584	0.5631	0.7830	0.075*	
C43	0.0837 (6)	0.6568 (3)	0.9081 (4)	0.030 (2)	
H43A	0.0622	0.6857	0.9186	0.044*	
H43B	0.1063	0.6578	0.8687	0.044*	
H43C	0.1282	0.6484	0.9355	0.044*	
C44	−0.0613 (7)	0.6356 (3)	0.8744 (5)	0.045 (3)	
H44A	−0.0445	0.6414	0.8343	0.067*	
H44B	−0.0882	0.6617	0.8904	0.067*	
H44C	−0.1007	0.6111	0.8751	0.067*	
C45	−0.0586 (6)	0.5189 (3)	0.9002 (4)	0.033 (2)	
H45A	−0.0893	0.5309	0.8668	0.050*	
H45B	−0.0841	0.4912	0.9120	0.050*	
H45C	−0.0001	0.5139	0.8895	0.050*	
C46	−0.1475 (6)	0.5588 (4)	0.9741 (5)	0.046 (3)	
H46A	−0.1706	0.5314	0.9890	0.069*	
H46B	−0.1843	0.5703	0.9435	0.069*	
H46C	−0.1433	0.5801	1.0059	0.069*	
C47	−0.0318 (7)	0.6524 (4)	1.0354 (5)	0.052 (3)	
H47A	0.0052	0.6701	1.0602	0.077*	
H47B	−0.0863	0.6491	1.0542	0.077*	
H47C	−0.0394	0.6669	0.9977	0.077*	
C48	0.0185 (7)	0.5820 (4)	1.0791 (4)	0.041 (3)	
H48A	−0.0361	0.5721	1.0931	0.061*	
H48B	0.0461	0.5996	1.1092	0.061*	
H48C	0.0534	0.5566	1.0700	0.061*	
Ce3	0.23763 (3)	0.863346 (13)	0.686301 (18)	0.01293 (10)	
P9	0.01473 (14)	0.80796 (7)	0.69531 (10)	0.0180 (5)	
P10	0.29502 (15)	0.78355 (7)	0.55792 (9)	0.0196 (5)	
P11	0.47679 (12)	0.86406 (7)	0.72080 (8)	0.0199 (4)	
P12	0.17030 (13)	0.96496 (6)	0.78115 (9)	0.0179 (4)	
O21	0.0972 (4)	0.83402 (17)	0.6931 (2)	0.0201 (12)	
O22	0.2730 (4)	0.81275 (17)	0.6093 (2)	0.0233 (13)	
O23	0.3859 (3)	0.87277 (18)	0.7014 (2)	0.0210 (12)	
O24	0.2046 (3)	0.93110 (17)	0.7384 (2)	0.0194 (13)	
O25	0.1551 (4)	0.90120 (18)	0.5999 (2)	0.0234 (13)	
O26	0.2086 (4)	0.9464 (2)	0.5346 (3)	0.0366 (17)	
O27	0.2877 (4)	0.9215 (2)	0.6084 (3)	0.0260 (14)	
O28	0.2624 (4)	0.78910 (18)	0.7428 (3)	0.0235 (14)	
O29	0.2364 (5)	0.7803 (2)	0.8374 (3)	0.052 (2)	
O30	0.2351 (4)	0.8463 (2)	0.7985 (3)	0.0290 (15)	
N29	0.0034 (5)	0.7856 (2)	0.7597 (3)	0.0217 (16)	
N30	0.0141 (4)	0.7679 (2)	0.6462 (3)	0.0227 (16)	
N31	−0.0664 (4)	0.8413 (2)	0.6819 (3)	0.0210 (15)	
N32	0.3498 (5)	0.7412 (2)	0.5819 (3)	0.0259 (17)	
N33	0.2078 (5)	0.7666 (2)	0.5247 (3)	0.0267 (17)	
N34	0.3529 (5)	0.8096 (2)	0.5093 (3)	0.0316 (18)	
N35	0.4986 (4)	0.8159 (2)	0.7492 (3)	0.0243 (15)	
N36	0.5008 (4)	0.9005 (2)	0.7721 (3)	0.0343 (17)	
N37	0.5401 (4)	0.8667 (2)	0.6626 (3)	0.0224 (14)	
N38	0.1737 (4)	0.9510 (2)	0.8517 (3)	0.0273 (16)	
N39	0.2289 (4)	1.01016 (19)	0.7791 (3)	0.0248 (15)	
N40	0.0708 (4)	0.9750 (2)	0.7634 (3)	0.0214 (15)	
N41	0.2181 (4)	0.9240 (2)	0.5799 (3)	0.0197 (15)	
N42	0.2433 (5)	0.8031 (2)	0.7939 (3)	0.0237 (17)	
C49	0.0221 (7)	0.8101 (3)	0.8150 (4)	0.038 (2)	
H49A	0.0658	0.7947	0.8370	0.057*	
H49B	−0.0288	0.8119	0.8383	0.057*	
H49C	0.0415	0.8396	0.8055	0.057*	
C50	−0.0419 (6)	0.7440 (3)	0.7722 (4)	0.032 (2)	
H50A	−0.0943	0.7506	0.7922	0.048*	
H50B	−0.0067	0.7254	0.7970	0.048*	
H50C	−0.0543	0.7288	0.7357	0.048*	
C51	0.0822 (6)	0.7340 (3)	0.6479 (4)	0.026 (2)	
H51A	0.1076	0.7316	0.6095	0.039*	
H51B	0.0581	0.7057	0.6589	0.039*	
H51C	0.1250	0.7425	0.6766	0.039*	
C52	−0.0576 (6)	0.7556 (3)	0.6091 (4)	0.030 (2)	
H52A	−0.0774	0.7265	0.6202	0.045*	
H52B	−0.0402	0.7552	0.5683	0.045*	
H52C	−0.1028	0.7769	0.6139	0.045*	
C53	−0.1515 (5)	0.8334 (3)	0.7045 (4)	0.028 (2)	
H53A	−0.1845	0.8171	0.6756	0.043*	
H53B	−0.1788	0.8615	0.7123	0.043*	
H53C	−0.1477	0.8165	0.7406	0.043*	
C54	−0.0633 (5)	0.8695 (3)	0.6286 (3)	0.030 (2)	
H54A	−0.0912	0.8974	0.6365	0.044*	
H54B	−0.0919	0.8547	0.5963	0.044*	
H54C	−0.0048	0.8749	0.6183	0.044*	
C55	0.4142 (6)	0.7175 (3)	0.5472 (4)	0.057 (3)	
H55A	0.3894	0.6911	0.5304	0.085*	
H55B	0.4614	0.7095	0.5726	0.085*	
H55C	0.4341	0.7365	0.5160	0.085*	
C56	0.3221 (6)	0.7151 (3)	0.6343 (3)	0.027 (2)	
H56A	0.3710	0.7013	0.6528	0.040*	
H56B	0.2823	0.6925	0.6219	0.040*	
H56C	0.2951	0.7346	0.6622	0.040*	
C57	0.2000 (7)	0.7238 (3)	0.4941 (4)	0.039 (2)	
H57A	0.2013	0.7285	0.4520	0.058*	
H57B	0.1470	0.7099	0.5045	0.058*	
H57C	0.2468	0.7048	0.5056	0.058*	
C58	0.1435 (6)	0.7986 (3)	0.5087 (4)	0.041 (3)	
H58A	0.0883	0.7876	0.5200	0.061*	
H58B	0.1443	0.8034	0.4666	0.061*	
H58C	0.1547	0.8262	0.5289	0.061*	
C59	0.3469 (7)	0.8029 (4)	0.4453 (4)	0.065 (4)	
H59A	0.3966	0.7872	0.4319	0.097*	
H59B	0.3434	0.8313	0.4258	0.097*	
H59C	0.2967	0.7858	0.4360	0.097*	
C60	0.4314 (7)	0.8317 (4)	0.5298 (4)	0.071 (4)	
H60A	0.4360	0.8604	0.5112	0.106*	
H60B	0.4799	0.8138	0.5196	0.106*	
H60C	0.4296	0.8354	0.5721	0.106*	
C61	0.4952 (6)	0.7753 (3)	0.7119 (4)	0.036 (2)	
H61A	0.4382	0.7635	0.7118	0.054*	
H61B	0.5341	0.7534	0.7276	0.054*	
H61C	0.5111	0.7826	0.6720	0.054*	
C62	0.4790 (6)	0.8062 (3)	0.8113 (3)	0.046 (2)	
H62A	0.5185	0.7844	0.8263	0.069*	
H62B	0.4218	0.7948	0.8139	0.069*	
H62C	0.4838	0.8331	0.8342	0.069*	
C63	0.5904 (6)	0.9043 (3)	0.7933 (5)	0.066 (3)	
H63A	0.5913	0.9076	0.8357	0.099*	
H63B	0.6167	0.9299	0.7755	0.099*	
H63C	0.6214	0.8779	0.7826	0.099*	
C64	0.4474 (6)	0.9357 (3)	0.7886 (5)	0.066 (3)	
H64A	0.4689	0.9631	0.7725	0.098*	
H64B	0.4459	0.9378	0.8311	0.098*	
H64C	0.3907	0.9305	0.7735	0.098*	
C65	0.6288 (5)	0.8505 (3)	0.6650 (4)	0.0317 (19)	
H65A	0.6672	0.8755	0.6647	0.048*	
H65B	0.6395	0.8320	0.6311	0.048*	
H65C	0.6377	0.8336	0.7006	0.048*	
C66	0.5280 (5)	0.9029 (2)	0.6201 (3)	0.0290 (18)	
H66A	0.5428	0.8928	0.5811	0.043*	
H66B	0.5639	0.9276	0.6311	0.043*	
H66C	0.4693	0.9122	0.6202	0.043*	
C67	0.1048 (6)	0.9256 (3)	0.8791 (4)	0.049 (3)	
H67A	0.1037	0.9318	0.9209	0.074*	
H67B	0.1143	0.8943	0.8730	0.074*	
H67C	0.0511	0.9340	0.8614	0.074*	
C68	0.2561 (6)	0.9414 (3)	0.8794 (4)	0.044 (2)	
H68A	0.2700	0.9106	0.8736	0.066*	
H68B	0.2534	0.9476	0.9211	0.066*	
H68C	0.2993	0.9597	0.8617	0.066*	
C69	0.2304 (6)	1.0442 (3)	0.8258 (4)	0.041 (2)	
H69A	0.1946	1.0687	0.8142	0.061*	
H69B	0.2879	1.0545	0.8318	0.061*	
H69C	0.2095	1.0315	0.8620	0.061*	
C70	0.2677 (6)	1.0235 (3)	0.7241 (4)	0.042 (2)	
H70A	0.3254	1.0330	0.7316	0.063*	
H70B	0.2357	1.0476	0.7069	0.063*	
H70C	0.2678	0.9987	0.6971	0.063*	
C71	0.0247 (6)	1.0142 (3)	0.7858 (4)	0.043 (2)	
H71A	0.0027	1.0078	0.8245	0.065*	
H71B	−0.0218	1.0212	0.7593	0.065*	
H71C	0.0631	1.0391	0.7882	0.065*	
C72	0.0136 (5)	0.9415 (3)	0.7384 (4)	0.034 (2)	
H72A	−0.0250	0.9553	0.7106	0.052*	
H72B	−0.0184	0.9278	0.7697	0.052*	
H72C	0.0465	0.9191	0.7183	0.052*	
Ce4	0.23742 (3)	0.868253 (13)	0.200816 (19)	0.01393 (10)	
P13	0.20401 (14)	0.98002 (7)	0.28357 (9)	0.0198 (4)	
P14	0.46704 (12)	0.85359 (6)	0.25212 (8)	0.0172 (4)	
P15	0.30069 (14)	0.80210 (7)	0.06128 (9)	0.0204 (5)	
P16	0.01329 (14)	0.81818 (7)	0.20925 (9)	0.0180 (5)	
O31	0.2193 (4)	0.93515 (18)	0.2576 (2)	0.0232 (13)	
O32	0.3845 (3)	0.86956 (18)	0.2254 (2)	0.0227 (13)	
O33	0.2713 (4)	0.82408 (18)	0.1174 (2)	0.0238 (14)	
O34	0.0944 (4)	0.84516 (17)	0.2086 (2)	0.0244 (13)	
O35	0.1552 (4)	0.9083 (2)	0.1148 (3)	0.0276 (14)	
O36	0.2185 (5)	0.9425 (2)	0.0417 (3)	0.0413 (18)	
O37	0.2903 (4)	0.9268 (2)	0.1230 (3)	0.0278 (15)	
O38	0.2289 (4)	0.83921 (18)	0.3096 (3)	0.0258 (14)	
O39	0.2178 (4)	0.76932 (19)	0.3336 (3)	0.0328 (16)	
O40	0.2506 (4)	0.78833 (18)	0.2426 (3)	0.0251 (14)	
N43	0.1124 (4)	0.9982 (2)	0.2576 (3)	0.0242 (15)	
N44	0.2709 (4)	1.0200 (2)	0.2678 (3)	0.0257 (16)	
N45	0.2122 (4)	0.9760 (2)	0.3557 (3)	0.0266 (15)	
N46	0.4608 (4)	0.8605 (2)	0.3254 (2)	0.0235 (14)	
N47	0.5449 (4)	0.8810 (2)	0.2212 (3)	0.0273 (15)	
N48	0.4928 (4)	0.8018 (2)	0.2459 (3)	0.0220 (15)	
N49	0.3577 (4)	0.8364 (2)	0.0225 (3)	0.0328 (17)	
N50	0.3590 (5)	0.7588 (2)	0.0769 (3)	0.0227 (16)	
N51	0.2195 (5)	0.7862 (2)	0.0203 (3)	0.0338 (19)	
N52	0.0059 (5)	0.7919 (2)	0.2716 (3)	0.0261 (17)	
N53	0.0128 (4)	0.7823 (2)	0.1551 (3)	0.0212 (16)	
N54	−0.0679 (4)	0.8521 (2)	0.2019 (3)	0.0242 (16)	
N55	0.2231 (5)	0.9268 (2)	0.0919 (3)	0.0220 (16)	
N56	0.2327 (4)	0.7973 (2)	0.2963 (3)	0.0195 (16)	
C73	0.0469 (5)	0.9664 (3)	0.2382 (4)	0.029 (2)	
H73A	0.0037	0.9817	0.2156	0.043*	
H73B	0.0214	0.9527	0.2722	0.043*	
H73C	0.0726	0.9438	0.2140	0.043*	
C74	0.0762 (6)	1.0408 (3)	0.2769 (4)	0.041 (2)	
H74A	0.0372	1.0357	0.3088	0.062*	
H74B	0.0462	1.0544	0.2442	0.062*	
H74C	0.1213	1.0601	0.2901	0.062*	
C75	0.2670 (7)	1.0431 (4)	0.2111 (4)	0.059 (3)	
H75A	0.3034	1.0283	0.1833	0.089*	
H75B	0.2859	1.0733	0.2163	0.089*	
H75C	0.2093	1.0429	0.1964	0.089*	
C76	0.3560 (6)	1.0217 (3)	0.2958 (4)	0.047 (2)	
H76A	0.3735	1.0522	0.3001	0.070*	
H76B	0.3963	1.0061	0.2716	0.070*	
H76C	0.3538	1.0080	0.3343	0.070*	
C77	0.2023 (6)	1.0147 (3)	0.3949 (3)	0.041 (2)	
H77A	0.2377	1.0110	0.4295	0.062*	
H77B	0.1436	1.0173	0.4065	0.062*	
H77C	0.2191	1.0412	0.3742	0.062*	
C78	0.1899 (6)	0.9336 (3)	0.3846 (4)	0.034 (2)	
H78A	0.1314	0.9345	0.3969	0.051*	
H78B	0.2263	0.9291	0.4186	0.051*	
H78C	0.1977	0.9095	0.3571	0.051*	
C79	0.4155 (5)	0.9006 (2)	0.3460 (3)	0.032 (2)	
H79A	0.4510	0.9263	0.3403	0.049*	
H79B	0.4026	0.8975	0.3874	0.049*	
H79C	0.3633	0.9041	0.3237	0.049*	
C80	0.5369 (5)	0.8506 (2)	0.3618 (3)	0.0275 (18)	
H80A	0.5202	0.8458	0.4022	0.041*	
H80B	0.5761	0.8752	0.3600	0.041*	
H80C	0.5642	0.8242	0.3469	0.041*	
C81	0.5308 (6)	0.9233 (3)	0.1934 (6)	0.091 (5)	
H81A	0.5648	0.9254	0.1583	0.136*	
H81B	0.5467	0.9467	0.2204	0.136*	
H81C	0.4715	0.9262	0.1829	0.136*	
C82	0.6350 (5)	0.8702 (3)	0.2298 (4)	0.039 (2)	
H82A	0.6617	0.8929	0.2538	0.058*	
H82B	0.6627	0.8688	0.1920	0.058*	
H82C	0.6401	0.8420	0.2495	0.058*	
C83	0.5227 (6)	0.7853 (3)	0.1878 (4)	0.038 (2)	
H83A	0.4757	0.7721	0.1664	0.057*	
H83B	0.5665	0.7633	0.1939	0.057*	
H83C	0.5454	0.8097	0.1653	0.057*	
C84	0.4576 (5)	0.7669 (3)	0.2838 (4)	0.0322 (19)	
H84A	0.5020	0.7462	0.2944	0.048*	
H84B	0.4128	0.7516	0.2629	0.048*	
H84C	0.4351	0.7802	0.3192	0.048*	
C85	0.4271 (6)	0.8621 (3)	0.0514 (4)	0.050 (3)	
H85A	0.4231	0.8928	0.0397	0.074*	
H85B	0.4814	0.8502	0.0395	0.074*	
H85C	0.4219	0.8599	0.0937	0.074*	
C86	0.3654 (7)	0.8349 (4)	−0.0419 (4)	0.064 (3)	
H86A	0.4149	0.8178	−0.0523	0.096*	
H86B	0.3709	0.8646	−0.0571	0.096*	
H86C	0.3153	0.8212	−0.0588	0.096*	
C87	0.4330 (5)	0.7455 (3)	0.0402 (4)	0.038 (2)	
H87A	0.4135	0.7269	0.0081	0.057*	
H87B	0.4734	0.7293	0.0642	0.057*	
H87C	0.4598	0.7716	0.0244	0.057*	
C88	0.3298 (6)	0.7254 (3)	0.1196 (4)	0.034 (2)	
H88A	0.3780	0.7137	0.1412	0.051*	
H88B	0.3015	0.7015	0.0988	0.051*	
H88C	0.2906	0.7389	0.1468	0.051*	
C89	0.1507 (7)	0.8183 (4)	0.0084 (5)	0.056 (3)	
H89A	0.0963	0.8041	0.0136	0.084*	
H89B	0.1550	0.8290	−0.0316	0.084*	
H89C	0.1557	0.8429	0.0355	0.084*	
C90	0.2130 (7)	0.7446 (3)	−0.0109 (4)	0.043 (3)	
H90A	0.2112	0.7502	−0.0529	0.064*	
H90B	0.1616	0.7295	0.0006	0.064*	
H90C	0.2616	0.7264	−0.0015	0.064*	
C91	−0.0344 (7)	0.7471 (3)	0.2791 (4)	0.038 (3)	
H91A	−0.0888	0.7504	0.2981	0.058*	
H91B	0.0021	0.7286	0.3031	0.058*	
H91C	−0.0425	0.7335	0.2408	0.058*	
C92	0.0140 (7)	0.8152 (4)	0.3277 (4)	0.045 (3)	
H92A	0.0532	0.7994	0.3531	0.068*	
H92B	−0.0409	0.8168	0.3463	0.068*	
H92C	0.0352	0.8448	0.3209	0.068*	
C93	0.0824 (6)	0.7501 (3)	0.1500 (4)	0.040 (3)	
H93A	0.1067	0.7521	0.1111	0.060*	
H93B	0.0606	0.7206	0.1562	0.060*	
H93C	0.1255	0.7565	0.1793	0.060*	
C94	−0.0619 (6)	0.7700 (3)	0.1196 (4)	0.033 (2)	
H94A	−0.0814	0.7410	0.1314	0.049*	
H94B	−0.0468	0.7694	0.0784	0.049*	
H94C	−0.1066	0.7914	0.1256	0.049*	
C95	−0.1524 (5)	0.8425 (3)	0.2243 (4)	0.040 (3)	
H95A	−0.1882	0.8320	0.1924	0.060*	
H95B	−0.1765	0.8692	0.2407	0.060*	
H95C	−0.1485	0.8200	0.2545	0.060*	
C96	−0.0648 (5)	0.8879 (3)	0.1576 (4)	0.038 (2)	
H96A	−0.0902	0.9144	0.1737	0.056*	
H96B	−0.0958	0.8790	0.1226	0.056*	
H96C	−0.0064	0.8939	0.1476	0.056*	

### Atomic displacement parameters (Å^2^)

**Table:**

	*U*^11^	*U*^22^	*U*^33^	*U*^12^	*U*^13^	*U*^23^
W1	0.01705 (18)	0.01836 (19)	0.01421 (16)	−0.00015 (12)	0.00113 (13)	−0.00098 (13)
W2	0.01651 (18)	0.02086 (19)	0.01315 (16)	0.00243 (13)	0.00137 (13)	0.00264 (13)
W3	0.01681 (18)	0.01789 (19)	0.01512 (17)	−0.00130 (12)	0.00127 (13)	−0.00188 (13)
W4	0.01607 (18)	0.01805 (19)	0.01398 (16)	−0.00043 (12)	0.00208 (13)	0.00047 (13)
Ag1	0.0426 (5)	0.0317 (4)	0.0156 (3)	0.0019 (3)	0.0014 (3)	0.0021 (3)
Ag2	0.0432 (5)	0.0348 (4)	0.0155 (3)	0.0027 (3)	0.0013 (3)	−0.0010 (3)
Ag3	0.0446 (5)	0.0320 (4)	0.0154 (3)	−0.0002 (3)	0.0013 (3)	0.0000 (3)
Ag4	0.0462 (5)	0.0337 (4)	0.0150 (3)	−0.0002 (3)	0.0013 (3)	−0.0017 (3)
S1	0.0197 (12)	0.0375 (12)	0.0225 (11)	−0.0053 (9)	0.0052 (9)	−0.0048 (10)
S2	0.0213 (12)	0.0328 (11)	0.0191 (10)	0.0025 (9)	−0.0006 (8)	0.0004 (9)
S3	0.0328 (13)	0.0169 (10)	0.0224 (11)	0.0026 (9)	0.0010 (9)	−0.0014 (9)
S4	0.0275 (13)	0.0220 (11)	0.0204 (11)	0.0015 (9)	−0.0002 (9)	−0.0053 (9)
S5	0.0196 (12)	0.0491 (14)	0.0226 (11)	0.0073 (10)	0.0047 (9)	0.0046 (11)
S6	0.0196 (12)	0.0343 (12)	0.0247 (11)	0.0004 (9)	−0.0025 (9)	0.0025 (10)
S7	0.0307 (13)	0.0216 (10)	0.0188 (10)	0.0024 (9)	−0.0001 (9)	0.0048 (9)
S8	0.0366 (14)	0.0225 (11)	0.0246 (12)	0.0028 (9)	−0.0016 (10)	0.0053 (10)
S9	0.0209 (12)	0.0370 (12)	0.0202 (11)	−0.0084 (9)	0.0055 (9)	−0.0019 (10)
S10	0.0217 (11)	0.0280 (11)	0.0211 (10)	−0.0009 (9)	−0.0004 (8)	0.0008 (9)
S11	0.0254 (12)	0.0159 (10)	0.0230 (11)	0.0006 (8)	0.0013 (9)	−0.0010 (9)
S12	0.0275 (12)	0.0218 (10)	0.0196 (10)	−0.0014 (9)	0.0015 (9)	−0.0074 (9)
S13	0.0214 (12)	0.0288 (11)	0.0207 (11)	0.0057 (9)	0.0061 (9)	0.0018 (9)
S14	0.0221 (12)	0.0349 (12)	0.0227 (11)	−0.0067 (9)	−0.0009 (9)	−0.0012 (10)
S15	0.0262 (12)	0.0240 (11)	0.0229 (11)	−0.0002 (9)	0.0039 (9)	0.0058 (9)
S16	0.0277 (12)	0.0238 (11)	0.0176 (10)	0.0011 (9)	0.0028 (8)	0.0004 (9)
Ce1	0.0114 (2)	0.0133 (2)	0.0156 (2)	−0.00090 (16)	−0.00033 (16)	0.00136 (19)
P1	0.0173 (11)	0.0195 (10)	0.0222 (10)	−0.0007 (8)	0.0019 (8)	0.0046 (9)
P2	0.0174 (11)	0.0353 (12)	0.0277 (11)	−0.0073 (9)	−0.0052 (8)	0.0077 (10)
P3	0.0264 (12)	0.0208 (10)	0.0215 (11)	0.0007 (9)	0.0057 (9)	0.0032 (9)
P4	0.0156 (11)	0.0182 (10)	0.0205 (11)	0.0002 (8)	0.0005 (8)	−0.0034 (9)
O1	0.020 (3)	0.015 (3)	0.019 (3)	−0.002 (2)	0.003 (2)	0.006 (2)
O2	0.013 (3)	0.025 (3)	0.034 (3)	−0.002 (2)	−0.004 (2)	0.003 (3)
O3	0.024 (3)	0.019 (3)	0.018 (3)	−0.004 (2)	0.005 (2)	0.005 (2)
O4	0.008 (3)	0.022 (3)	0.032 (3)	−0.002 (2)	−0.004 (2)	0.005 (2)
O5	0.036 (4)	0.026 (3)	0.030 (3)	−0.006 (3)	0.000 (3)	0.002 (3)
O6	0.127 (8)	0.041 (4)	0.046 (4)	−0.028 (5)	0.015 (5)	−0.023 (4)
O7	0.042 (4)	0.019 (3)	0.029 (3)	−0.006 (3)	−0.007 (3)	0.008 (3)
O8	0.016 (3)	0.024 (3)	0.029 (3)	−0.003 (2)	0.001 (2)	−0.001 (2)
O9	0.039 (4)	0.026 (3)	0.033 (4)	−0.007 (3)	0.002 (3)	−0.006 (3)
O10	0.019 (3)	0.018 (3)	0.027 (3)	0.003 (2)	−0.003 (2)	−0.003 (2)
N1	0.014 (4)	0.030 (4)	0.038 (4)	−0.007 (3)	0.000 (3)	0.014 (3)
N2	0.047 (5)	0.029 (4)	0.023 (4)	−0.008 (3)	0.010 (3)	−0.008 (3)
N3	0.023 (4)	0.023 (3)	0.024 (3)	0.006 (3)	−0.002 (3)	0.000 (3)
N4	0.036 (5)	0.070 (6)	0.049 (5)	−0.008 (4)	0.001 (4)	−0.018 (5)
N5	0.018 (4)	0.079 (6)	0.064 (5)	0.008 (4)	0.000 (4)	0.045 (5)
N6	0.012 (4)	0.039 (4)	0.050 (4)	−0.005 (3)	0.005 (3)	0.002 (4)
N7	0.024 (4)	0.023 (3)	0.030 (4)	−0.003 (3)	0.001 (3)	−0.004 (3)
N8	0.050 (5)	0.036 (4)	0.023 (4)	0.014 (3)	0.023 (3)	0.010 (3)
N9	0.023 (4)	0.026 (4)	0.023 (4)	0.000 (3)	0.005 (3)	−0.002 (3)
N10	0.015 (4)	0.018 (3)	0.026 (4)	0.001 (3)	−0.004 (3)	0.003 (3)
N11	0.018 (4)	0.019 (3)	0.023 (3)	0.000 (3)	−0.001 (3)	−0.002 (3)
N12	0.032 (4)	0.021 (4)	0.033 (4)	0.002 (3)	0.002 (3)	−0.008 (3)
N13	0.026 (4)	0.014 (3)	0.021 (4)	−0.005 (3)	−0.001 (3)	−0.002 (3)
N14	0.045 (6)	0.028 (4)	0.045 (5)	−0.002 (4)	−0.002 (4)	−0.021 (4)
C1	0.019 (5)	0.037 (5)	0.042 (5)	0.004 (4)	0.004 (4)	−0.002 (4)
C2	0.033 (6)	0.059 (6)	0.085 (8)	−0.019 (5)	0.000 (5)	0.038 (6)
C3	0.078 (10)	0.212 (17)	0.026 (6)	0.052 (10)	0.006 (6)	−0.031 (8)
C4	0.063 (8)	0.083 (8)	0.024 (5)	−0.025 (6)	−0.006 (5)	−0.015 (5)
C5	0.051 (6)	0.024 (4)	0.055 (6)	−0.001 (4)	−0.008 (5)	0.014 (4)
C6	0.026 (5)	0.026 (4)	0.046 (5)	0.008 (4)	−0.006 (4)	−0.013 (4)
C7	0.036 (6)	0.066 (8)	0.110 (10)	0.028 (5)	−0.040 (6)	−0.060 (8)
C8	0.098 (11)	0.209 (17)	0.048 (7)	−0.080 (11)	0.030 (7)	−0.065 (9)
C9	0.034 (5)	0.029 (4)	0.055 (6)	−0.004 (4)	−0.011 (4)	0.004 (4)
C10	0.026 (6)	0.146 (12)	0.103 (9)	0.008 (7)	0.001 (6)	0.080 (9)
C11	0.034 (6)	0.063 (7)	0.078 (7)	−0.015 (5)	0.012 (5)	−0.003 (6)
C12	0.048 (6)	0.053 (6)	0.039 (5)	−0.002 (5)	0.014 (4)	−0.001 (5)
C13	0.038 (6)	0.016 (4)	0.030 (5)	−0.009 (4)	0.005 (4)	−0.008 (4)
C14	0.035 (5)	0.033 (5)	0.069 (6)	−0.004 (4)	0.006 (5)	0.004 (5)
C15	0.061 (6)	0.033 (4)	0.027 (4)	−0.014 (4)	0.033 (4)	−0.008 (4)
C16	0.073 (8)	0.043 (5)	0.042 (5)	0.018 (5)	0.029 (5)	0.016 (5)
C17	0.035 (5)	0.031 (5)	0.024 (4)	0.002 (4)	−0.005 (4)	0.004 (4)
C18	0.038 (6)	0.041 (6)	0.039 (5)	0.004 (5)	−0.008 (4)	−0.004 (5)
C19	0.031 (5)	0.019 (4)	0.035 (5)	−0.005 (4)	0.000 (4)	0.000 (4)
C20	0.030 (5)	0.032 (5)	0.023 (4)	0.001 (4)	−0.007 (4)	0.005 (4)
C21	0.026 (5)	0.021 (4)	0.033 (4)	−0.001 (3)	−0.004 (4)	−0.011 (4)
C22	0.018 (5)	0.042 (5)	0.048 (6)	0.003 (4)	−0.004 (4)	0.009 (5)
C23	0.036 (6)	0.035 (5)	0.039 (5)	−0.001 (4)	0.006 (4)	−0.019 (4)
C24	0.037 (6)	0.033 (5)	0.028 (5)	0.001 (4)	−0.001 (4)	−0.005 (4)
Ce2	0.0105 (2)	0.0135 (2)	0.0153 (2)	−0.00026 (16)	0.00115 (16)	0.00153 (18)
P5	0.0228 (12)	0.0178 (10)	0.0198 (10)	−0.0028 (8)	−0.0006 (8)	0.0031 (9)
P6	0.0131 (10)	0.0177 (10)	0.0217 (10)	−0.0017 (7)	−0.0006 (8)	−0.0003 (8)
P7	0.0211 (11)	0.0218 (10)	0.0155 (10)	0.0000 (8)	0.0037 (8)	0.0041 (9)
P8	0.0109 (11)	0.0202 (10)	0.0258 (11)	0.0000 (8)	0.0018 (8)	−0.0042 (9)
O11	0.023 (3)	0.016 (3)	0.014 (3)	0.001 (2)	0.002 (2)	0.001 (2)
O12	0.011 (3)	0.026 (3)	0.024 (3)	−0.004 (2)	−0.007 (2)	0.002 (2)
O13	0.020 (3)	0.019 (3)	0.023 (3)	−0.002 (2)	0.001 (2)	0.006 (2)
O14	0.007 (3)	0.023 (3)	0.022 (3)	−0.001 (2)	0.004 (2)	−0.001 (2)
O15	0.012 (3)	0.026 (3)	0.027 (3)	−0.004 (2)	0.008 (2)	−0.004 (2)
O16	0.055 (5)	0.048 (4)	0.023 (3)	−0.007 (3)	0.009 (3)	−0.019 (3)
O17	0.021 (3)	0.026 (3)	0.032 (3)	0.008 (2)	0.001 (3)	−0.005 (3)
O18	0.023 (3)	0.024 (3)	0.020 (3)	0.002 (2)	0.001 (2)	0.002 (3)
O19	0.071 (5)	0.036 (4)	0.030 (4)	−0.018 (3)	0.011 (3)	−0.027 (3)
O20	0.032 (4)	0.022 (3)	0.021 (3)	−0.001 (3)	0.004 (3)	0.005 (3)
N15	0.023 (4)	0.021 (3)	0.047 (4)	−0.007 (3)	−0.009 (3)	0.007 (3)
N16	0.029 (4)	0.024 (3)	0.034 (4)	−0.004 (3)	0.002 (3)	0.012 (3)
N17	0.021 (4)	0.023 (4)	0.040 (4)	0.003 (3)	−0.005 (3)	−0.004 (3)
N18	0.021 (3)	0.022 (3)	0.028 (3)	−0.004 (3)	−0.005 (3)	0.009 (3)
N19	0.013 (4)	0.041 (4)	0.048 (4)	0.000 (3)	−0.004 (3)	−0.030 (4)
N20	0.028 (4)	0.014 (3)	0.025 (3)	−0.004 (3)	−0.007 (3)	0.000 (3)
N21	0.040 (5)	0.038 (4)	0.017 (3)	0.001 (3)	0.013 (3)	−0.007 (3)
N22	0.030 (4)	0.024 (4)	0.024 (4)	−0.007 (3)	0.003 (3)	−0.001 (3)
N23	0.026 (4)	0.035 (4)	0.031 (4)	−0.006 (3)	−0.002 (3)	0.009 (3)
N24	0.018 (4)	0.019 (3)	0.030 (4)	0.005 (3)	−0.009 (3)	−0.001 (3)
N25	0.015 (4)	0.028 (4)	0.040 (4)	0.002 (3)	0.001 (3)	−0.010 (3)
N26	0.025 (4)	0.026 (4)	0.019 (4)	0.002 (3)	0.005 (3)	−0.006 (3)
N27	0.045 (5)	0.021 (4)	0.020 (4)	−0.007 (3)	0.010 (3)	−0.003 (3)
N28	0.027 (5)	0.044 (5)	0.025 (4)	−0.007 (4)	0.006 (3)	−0.007 (4)
C25	0.013 (5)	0.050 (6)	0.045 (5)	0.004 (4)	0.004 (4)	0.005 (5)
C26	0.036 (6)	0.038 (5)	0.103 (8)	−0.019 (5)	−0.027 (6)	0.015 (6)
C27	0.054 (6)	0.042 (5)	0.034 (5)	−0.006 (4)	0.004 (4)	0.012 (4)
C28	0.064 (7)	0.036 (5)	0.023 (5)	−0.002 (5)	0.005 (5)	−0.005 (4)
C29	0.043 (6)	0.038 (5)	0.051 (5)	0.014 (4)	−0.009 (4)	−0.004 (5)
C30	0.064 (8)	0.077 (8)	0.063 (7)	0.022 (6)	−0.015 (6)	−0.037 (7)
C31	0.031 (5)	0.035 (4)	0.034 (4)	−0.011 (4)	−0.009 (4)	0.018 (4)
C32	0.035 (5)	0.043 (5)	0.036 (4)	0.000 (4)	−0.011 (4)	0.010 (4)
C33	0.017 (4)	0.042 (5)	0.064 (6)	0.008 (4)	−0.008 (4)	−0.034 (5)
C34	0.025 (5)	0.071 (7)	0.054 (5)	0.003 (5)	−0.003 (4)	−0.037 (5)
C35	0.030 (5)	0.017 (4)	0.050 (5)	−0.001 (3)	−0.005 (4)	−0.007 (4)
C36	0.056 (7)	0.034 (5)	0.039 (5)	−0.018 (5)	−0.004 (5)	0.018 (4)
C37	0.035 (6)	0.042 (5)	0.081 (7)	0.015 (4)	0.028 (5)	−0.020 (5)
C38	0.075 (9)	0.079 (8)	0.037 (6)	−0.010 (6)	0.011 (5)	−0.027 (6)
C39	0.057 (7)	0.036 (5)	0.038 (5)	−0.015 (4)	0.020 (5)	−0.003 (4)
C40	0.039 (6)	0.031 (5)	0.041 (5)	−0.003 (4)	0.009 (4)	0.000 (4)
C41	0.043 (6)	0.040 (5)	0.030 (5)	0.010 (4)	0.004 (4)	0.014 (4)
C42	0.044 (7)	0.042 (5)	0.064 (7)	−0.022 (5)	−0.018 (5)	0.014 (5)
C43	0.022 (5)	0.024 (4)	0.043 (5)	−0.006 (3)	0.002 (4)	0.012 (4)
C44	0.035 (6)	0.029 (5)	0.069 (7)	0.013 (4)	−0.025 (5)	0.002 (5)
C45	0.037 (5)	0.017 (4)	0.046 (5)	0.002 (3)	−0.011 (4)	−0.006 (4)
C46	0.026 (6)	0.047 (6)	0.065 (7)	0.000 (4)	0.008 (5)	−0.008 (5)
C47	0.040 (7)	0.067 (7)	0.048 (6)	−0.012 (5)	0.019 (5)	−0.019 (6)
C48	0.029 (6)	0.060 (6)	0.033 (5)	0.000 (5)	0.013 (4)	0.005 (5)
Ce3	0.0116 (2)	0.0123 (2)	0.0149 (2)	0.00061 (16)	0.00046 (16)	−0.00151 (18)
P9	0.0115 (10)	0.0173 (10)	0.0251 (11)	−0.0015 (8)	0.0010 (8)	0.0005 (9)
P10	0.0236 (12)	0.0197 (10)	0.0156 (10)	−0.0034 (9)	0.0041 (8)	−0.0046 (9)
P11	0.0144 (10)	0.0231 (10)	0.0221 (10)	0.0019 (8)	−0.0021 (8)	−0.0052 (9)
P12	0.0200 (11)	0.0144 (9)	0.0192 (10)	0.0026 (8)	0.0024 (8)	−0.0029 (8)
O21	0.018 (3)	0.021 (3)	0.022 (3)	−0.001 (2)	0.002 (2)	0.002 (2)
O22	0.029 (3)	0.016 (3)	0.025 (3)	0.000 (2)	0.003 (2)	−0.004 (2)
O23	0.007 (3)	0.031 (3)	0.025 (3)	−0.001 (2)	−0.003 (2)	−0.003 (3)
O24	0.018 (3)	0.018 (3)	0.023 (3)	0.003 (2)	0.000 (2)	−0.008 (2)
O25	0.022 (3)	0.018 (3)	0.030 (3)	−0.007 (2)	−0.006 (2)	0.008 (2)
O26	0.034 (4)	0.050 (4)	0.026 (3)	0.004 (3)	−0.001 (3)	0.022 (3)
O27	0.020 (3)	0.033 (3)	0.025 (3)	−0.006 (2)	0.003 (3)	0.002 (3)
O28	0.032 (4)	0.017 (3)	0.021 (3)	0.011 (2)	0.005 (2)	−0.001 (2)
O29	0.087 (6)	0.040 (4)	0.030 (4)	0.020 (4)	0.012 (4)	0.018 (3)
O30	0.041 (4)	0.023 (3)	0.024 (3)	0.006 (3)	0.005 (3)	0.000 (3)
N29	0.026 (4)	0.027 (4)	0.012 (3)	−0.002 (3)	0.005 (3)	0.002 (3)
N30	0.010 (4)	0.031 (4)	0.027 (4)	0.000 (3)	−0.002 (3)	−0.004 (3)
N31	0.015 (4)	0.019 (3)	0.029 (4)	0.001 (3)	0.005 (3)	0.004 (3)
N32	0.028 (4)	0.025 (4)	0.025 (4)	0.008 (3)	0.007 (3)	−0.012 (3)
N33	0.027 (4)	0.024 (4)	0.028 (4)	−0.002 (3)	−0.003 (3)	−0.014 (3)
N34	0.034 (4)	0.040 (4)	0.020 (3)	−0.021 (3)	0.003 (3)	−0.006 (3)
N35	0.029 (4)	0.021 (3)	0.023 (3)	0.002 (3)	0.007 (3)	0.002 (3)
N36	0.014 (3)	0.043 (4)	0.046 (4)	0.009 (3)	−0.013 (3)	−0.030 (3)
N37	0.017 (3)	0.023 (3)	0.027 (3)	0.001 (3)	−0.003 (3)	0.006 (3)
N38	0.026 (4)	0.037 (4)	0.019 (3)	0.001 (3)	0.005 (3)	−0.004 (3)
N39	0.026 (4)	0.011 (3)	0.037 (4)	0.001 (3)	0.002 (3)	−0.009 (3)
N40	0.024 (4)	0.015 (3)	0.025 (3)	0.007 (3)	−0.002 (3)	−0.003 (3)
N41	0.018 (4)	0.023 (3)	0.019 (4)	0.002 (3)	0.002 (3)	0.001 (3)
N42	0.034 (4)	0.021 (4)	0.016 (3)	0.009 (3)	−0.003 (3)	0.002 (3)
C49	0.053 (7)	0.037 (5)	0.024 (5)	−0.009 (5)	−0.001 (4)	0.001 (4)
C50	0.039 (6)	0.014 (4)	0.043 (5)	−0.005 (4)	0.017 (4)	0.011 (4)
C51	0.023 (5)	0.019 (4)	0.037 (5)	0.000 (3)	−0.001 (4)	−0.003 (4)
C52	0.013 (4)	0.031 (5)	0.047 (5)	0.000 (3)	0.000 (4)	−0.005 (4)
C53	0.017 (5)	0.027 (4)	0.041 (5)	0.007 (3)	0.009 (4)	−0.006 (4)
C54	0.017 (4)	0.031 (5)	0.040 (5)	0.006 (3)	−0.011 (4)	−0.006 (4)
C55	0.041 (6)	0.070 (7)	0.059 (6)	0.023 (5)	0.017 (5)	−0.020 (6)
C56	0.044 (6)	0.013 (4)	0.023 (4)	−0.010 (4)	−0.005 (4)	0.001 (4)
C57	0.043 (6)	0.033 (5)	0.040 (5)	−0.006 (4)	0.003 (4)	−0.030 (5)
C58	0.031 (6)	0.049 (6)	0.043 (6)	0.001 (5)	−0.001 (4)	−0.008 (5)
C59	0.080 (9)	0.095 (9)	0.019 (5)	−0.053 (7)	0.003 (5)	−0.003 (5)
C60	0.088 (9)	0.092 (8)	0.032 (5)	−0.058 (7)	0.007 (5)	−0.005 (5)
C61	0.037 (5)	0.026 (4)	0.046 (5)	0.001 (4)	−0.003 (4)	0.002 (4)
C62	0.051 (6)	0.057 (6)	0.030 (5)	0.021 (5)	0.004 (4)	0.025 (4)
C63	0.035 (6)	0.072 (7)	0.090 (8)	0.007 (5)	−0.025 (5)	−0.060 (6)
C64	0.035 (6)	0.078 (8)	0.083 (8)	0.009 (5)	−0.017 (5)	−0.049 (7)
C65	0.021 (5)	0.031 (4)	0.044 (5)	0.003 (4)	0.006 (4)	0.005 (4)
C66	0.018 (4)	0.032 (4)	0.037 (4)	−0.001 (3)	0.000 (3)	0.012 (4)
C67	0.046 (6)	0.063 (6)	0.038 (5)	−0.020 (5)	0.000 (4)	0.010 (5)
C68	0.058 (7)	0.050 (6)	0.023 (5)	0.002 (5)	−0.007 (4)	0.001 (4)
C69	0.043 (6)	0.024 (4)	0.055 (5)	0.000 (4)	−0.007 (4)	−0.014 (4)
C70	0.032 (6)	0.037 (5)	0.057 (6)	−0.010 (4)	−0.001 (5)	0.009 (5)
C71	0.025 (5)	0.037 (5)	0.068 (6)	0.008 (4)	0.000 (4)	−0.024 (5)
C72	0.026 (5)	0.039 (5)	0.038 (5)	0.008 (4)	−0.001 (4)	0.003 (4)
Ce4	0.0111 (2)	0.0124 (2)	0.0183 (2)	0.00022 (16)	0.00083 (16)	−0.00175 (18)
P13	0.0233 (12)	0.0144 (10)	0.0216 (10)	0.0010 (8)	0.0041 (8)	−0.0045 (9)
P14	0.0120 (10)	0.0173 (9)	0.0222 (10)	−0.0002 (7)	−0.0003 (8)	0.0010 (8)
P15	0.0228 (11)	0.0171 (10)	0.0215 (10)	0.0014 (8)	0.0036 (8)	−0.0009 (9)
P16	0.0119 (11)	0.0187 (10)	0.0234 (11)	−0.0017 (8)	0.0018 (8)	0.0004 (9)
O31	0.026 (3)	0.020 (3)	0.023 (3)	0.004 (2)	0.006 (2)	−0.004 (2)
O32	0.014 (3)	0.024 (3)	0.030 (3)	0.002 (2)	−0.004 (2)	0.000 (3)
O33	0.026 (3)	0.028 (3)	0.018 (3)	0.006 (2)	0.005 (2)	−0.015 (2)
O34	0.022 (3)	0.017 (3)	0.034 (3)	−0.006 (2)	−0.002 (2)	−0.002 (2)
O35	0.016 (3)	0.033 (3)	0.034 (3)	0.001 (2)	0.004 (2)	0.012 (3)
O36	0.051 (5)	0.040 (4)	0.033 (4)	0.001 (3)	0.005 (3)	0.016 (3)
O37	0.027 (4)	0.029 (3)	0.027 (3)	−0.009 (3)	0.006 (3)	0.000 (3)
O38	0.030 (4)	0.017 (3)	0.030 (3)	0.003 (2)	−0.003 (3)	0.002 (3)
O39	0.054 (4)	0.018 (3)	0.026 (3)	−0.002 (3)	0.001 (3)	0.007 (3)
O40	0.033 (4)	0.013 (3)	0.029 (3)	0.007 (2)	0.000 (3)	−0.007 (3)
N43	0.027 (4)	0.018 (3)	0.028 (3)	0.005 (3)	−0.001 (3)	−0.007 (3)
N44	0.036 (4)	0.014 (3)	0.027 (4)	−0.001 (3)	0.007 (3)	0.006 (3)
N45	0.040 (4)	0.019 (3)	0.021 (3)	−0.002 (3)	0.009 (3)	−0.007 (3)
N46	0.016 (3)	0.030 (3)	0.024 (3)	0.008 (3)	−0.006 (2)	−0.011 (3)
N47	0.009 (3)	0.034 (4)	0.039 (4)	0.001 (3)	0.001 (3)	0.023 (3)
N48	0.025 (4)	0.020 (3)	0.021 (3)	−0.002 (3)	0.007 (3)	0.003 (3)
N49	0.033 (4)	0.036 (4)	0.029 (4)	0.003 (3)	0.010 (3)	0.003 (3)
N50	0.029 (4)	0.019 (3)	0.021 (3)	−0.002 (3)	0.012 (3)	0.001 (3)
N51	0.037 (5)	0.035 (4)	0.029 (4)	0.000 (3)	−0.005 (3)	−0.012 (3)
N52	0.028 (4)	0.029 (4)	0.021 (4)	−0.006 (3)	−0.004 (3)	0.004 (3)
N53	0.017 (4)	0.028 (4)	0.020 (3)	0.000 (3)	0.001 (3)	−0.003 (3)
N54	0.015 (4)	0.022 (3)	0.036 (4)	0.003 (3)	0.003 (3)	0.002 (3)
N55	0.024 (4)	0.017 (3)	0.025 (4)	0.003 (3)	−0.001 (3)	−0.001 (3)
N56	0.017 (4)	0.014 (3)	0.027 (4)	0.002 (3)	−0.010 (3)	−0.001 (3)
C73	0.018 (5)	0.032 (5)	0.035 (5)	0.009 (4)	0.000 (4)	0.003 (4)
C74	0.046 (6)	0.028 (5)	0.051 (5)	0.019 (4)	−0.004 (4)	−0.007 (4)
C75	0.057 (7)	0.066 (7)	0.055 (6)	−0.015 (6)	0.004 (5)	0.035 (6)
C76	0.031 (5)	0.036 (5)	0.073 (6)	−0.008 (4)	−0.008 (5)	0.010 (5)
C77	0.059 (7)	0.030 (5)	0.035 (5)	0.006 (4)	−0.002 (4)	−0.013 (4)
C78	0.048 (6)	0.027 (5)	0.028 (5)	0.004 (4)	0.010 (4)	0.001 (4)
C79	0.031 (5)	0.033 (4)	0.033 (4)	0.011 (4)	0.003 (4)	−0.017 (4)
C80	0.023 (4)	0.026 (4)	0.034 (4)	0.006 (3)	−0.008 (3)	0.000 (4)
C81	0.033 (6)	0.061 (7)	0.179 (13)	−0.001 (5)	0.014 (7)	0.081 (8)
C82	0.007 (4)	0.044 (5)	0.065 (6)	0.002 (4)	0.001 (4)	0.027 (5)
C83	0.043 (6)	0.036 (5)	0.037 (5)	0.013 (4)	0.002 (4)	−0.012 (4)
C84	0.026 (5)	0.028 (4)	0.043 (5)	−0.001 (4)	−0.004 (4)	0.006 (4)
C85	0.046 (6)	0.040 (5)	0.063 (6)	−0.022 (4)	0.029 (5)	−0.012 (5)
C86	0.066 (8)	0.092 (9)	0.034 (5)	0.012 (6)	0.015 (5)	0.025 (6)
C87	0.033 (5)	0.036 (5)	0.045 (5)	0.009 (4)	0.010 (4)	0.000 (4)
C88	0.042 (6)	0.021 (5)	0.039 (5)	−0.003 (4)	0.010 (4)	−0.005 (4)
C89	0.040 (6)	0.068 (8)	0.059 (7)	0.008 (5)	−0.021 (5)	−0.017 (6)
C90	0.058 (7)	0.043 (6)	0.028 (5)	−0.006 (5)	0.005 (4)	−0.017 (5)
C91	0.047 (7)	0.022 (5)	0.046 (6)	−0.023 (4)	0.010 (5)	0.005 (4)
C92	0.056 (7)	0.049 (6)	0.031 (5)	−0.013 (5)	0.000 (5)	0.003 (5)
C93	0.041 (6)	0.042 (6)	0.036 (5)	−0.004 (5)	−0.011 (4)	−0.008 (5)
C94	0.026 (5)	0.038 (5)	0.034 (5)	−0.009 (4)	−0.004 (4)	−0.003 (4)
C95	0.007 (4)	0.050 (6)	0.064 (7)	0.008 (4)	0.007 (4)	0.004 (5)
C96	0.014 (4)	0.041 (5)	0.057 (6)	0.005 (4)	−0.015 (4)	−0.001 (5)

### Geometric parameters (Å)

**Table:**

W1—S2	2.202 (2)	C42—H42A	0.9600
W1—S1	2.203 (2)	C42—H42B	0.9600
W1—S4	2.205 (2)	C42—H42C	0.9600
W1—S3	2.217 (2)	C43—H43A	0.9600
W1—Ag1	2.9307 (9)	C43—H43B	0.9600
W1—Ag4	2.9424 (9)	C43—H43C	0.9600
W2—S5	2.203 (2)	C44—H44A	0.9600
W2—S6	2.206 (2)	C44—H44B	0.9600
W2—S8	2.208 (2)	C44—H44C	0.9600
W2—S7	2.209 (2)	C45—H45A	0.9600
W2—Ag2	2.9320 (9)	C45—H45B	0.9600
W2—Ag1	2.9458 (9)	C45—H45C	0.9600
W3—S9	2.196 (2)	C46—H46A	0.9600
W3—S12	2.207 (2)	C46—H46B	0.9600
W3—S10	2.207 (2)	C46—H46C	0.9600
W3—S11	2.216 (2)	C47—H47A	0.9600
W3—Ag2	2.9300 (9)	C47—H47B	0.9600
W3—Ag3	2.9307 (9)	C47—H47C	0.9600
W4—S13	2.201 (2)	C48—H48A	0.9600
W4—S14	2.207 (2)	C48—H48B	0.9600
W4—S16	2.207 (2)	C48—H48C	0.9600
W4—S15	2.211 (2)	Ce3—O23	2.358 (5)
W4—Ag4^i^	2.9292 (9)	Ce3—O21	2.372 (6)
W4—Ag3	2.9402 (9)	Ce3—O22	2.375 (5)
Ag1—S3	2.479 (2)	Ce3—O24	2.406 (5)
Ag1—S4	2.572 (2)	Ce3—O30	2.580 (6)
Ag1—S6	2.605 (3)	Ce3—O25	2.589 (5)
Ag1—S5	2.626 (3)	Ce3—O28	2.593 (6)
Ag2—S8	2.480 (2)	Ce3—O27	2.599 (6)
Ag2—S7	2.571 (2)	Ce3—N41	3.023 (7)
Ag2—S9	2.600 (3)	Ce3—N42	3.025 (7)
Ag2—S10	2.602 (2)	P9—O21	1.510 (6)
Ag3—S11	2.476 (2)	P9—N29	1.610 (7)
Ag3—S12	2.568 (2)	P9—N30	1.633 (7)
Ag3—S14	2.616 (2)	P9—N31	1.641 (7)
Ag3—S13	2.650 (3)	P10—O22	1.494 (6)
Ag4—S16^ii^	2.487 (2)	P10—N32	1.624 (7)
Ag4—S15^ii^	2.577 (2)	P10—N34	1.627 (7)
Ag4—S2	2.633 (2)	P10—N33	1.630 (7)
Ag4—S1	2.643 (3)	P11—O23	1.506 (5)
Ag4—W4^ii^	2.9292 (9)	P11—N35	1.615 (6)
S15—Ag4^i^	2.577 (2)	P11—N36	1.632 (6)
S16—Ag4^i^	2.487 (2)	P11—N37	1.653 (6)
Ce1—O2	2.363 (5)	P12—O24	1.502 (5)
Ce1—O4	2.382 (5)	P12—N40	1.631 (7)
Ce1—O3	2.391 (5)	P12—N39	1.638 (6)
Ce1—O1	2.409 (5)	P12—N38	1.645 (7)
Ce1—O7	2.576 (6)	O25—N41	1.284 (9)
Ce1—O8	2.583 (6)	O26—N41	1.230 (9)
Ce1—O10	2.595 (5)	O27—N41	1.262 (9)
Ce1—O5	2.628 (6)	O28—N42	1.264 (9)
Ce1—N14	3.002 (8)	O29—N42	1.199 (9)
Ce1—N13	3.021 (7)	O30—N42	1.306 (9)
P1—O1	1.486 (5)	N29—C50	1.463 (10)
P1—N1	1.628 (7)	N29—C49	1.473 (11)
P1—N3	1.631 (6)	N30—C52	1.441 (10)
P1—N2	1.647 (7)	N30—C51	1.474 (10)
P2—O2	1.502 (6)	N31—C53	1.450 (10)
P2—N4	1.589 (8)	N31—C54	1.471 (10)
P2—N6	1.614 (7)	N32—C55	1.463 (10)
P2—N5	1.634 (7)	N32—C56	1.485 (10)
P3—O3	1.491 (5)	N33—C58	1.434 (12)
P3—N8	1.641 (7)	N33—C57	1.464 (10)
P3—N9	1.642 (7)	N34—C59	1.459 (10)
P3—N7	1.645 (7)	N34—C60	1.466 (11)
P4—O4	1.490 (6)	N35—C62	1.464 (10)
P4—N11	1.632 (7)	N35—C61	1.480 (10)
P4—N12	1.635 (7)	N36—C64	1.400 (10)
P4—N10	1.637 (7)	N36—C63	1.481 (10)
O5—N14	1.282 (10)	N37—C66	1.459 (9)
O6—N14	1.228 (10)	N37—C65	1.470 (9)
O7—N14	1.258 (10)	N38—C68	1.455 (11)
O8—N13	1.240 (9)	N38—C67	1.461 (10)
O9—N13	1.214 (9)	N39—C70	1.441 (10)
O10—N13	1.279 (9)	N39—C69	1.466 (9)
N1—C1	1.458 (10)	N40—C72	1.456 (10)
N1—C2	1.476 (10)	N40—C71	1.471 (9)
N2—C3	1.407 (12)	C49—H49A	0.9600
N2—C4	1.460 (11)	C49—H49B	0.9600
N3—C6	1.462 (9)	C49—H49C	0.9600
N3—C5	1.473 (9)	C50—H50A	0.9600
N4—C7	1.449 (13)	C50—H50B	0.9600
N4—C8	1.471 (12)	C50—H50C	0.9600
N5—C9	1.406 (10)	C51—H51A	0.9600
N5—C10	1.472 (11)	C51—H51B	0.9600
N6—C11	1.452 (10)	C51—H51C	0.9600
N6—C12	1.460 (10)	C52—H52A	0.9600
N7—C13	1.449 (10)	C52—H52B	0.9600
N7—C14	1.458 (11)	C52—H52C	0.9600
N8—C15	1.419 (10)	C53—H53A	0.9600
N8—C16	1.465 (10)	C53—H53B	0.9600
N9—C17	1.458 (10)	C53—H53C	0.9600
N9—C18	1.475 (11)	C54—H54A	0.9600
N10—C19	1.448 (11)	C54—H54B	0.9600
N10—C20	1.461 (10)	C54—H54C	0.9600
N11—C21	1.454 (9)	C55—H55A	0.9600
N11—C22	1.481 (11)	C55—H55B	0.9600
N12—C23	1.443 (11)	C55—H55C	0.9600
N12—C24	1.476 (11)	C56—H56A	0.9600
C1—H1A	0.9600	C56—H56B	0.9600
C1—H1B	0.9600	C56—H56C	0.9600
C1—H1C	0.9600	C57—H57A	0.9600
C2—H2A	0.9600	C57—H57B	0.9600
C2—H2B	0.9600	C57—H57C	0.9600
C2—H2C	0.9600	C58—H58A	0.9600
C3—H3A	0.9600	C58—H58B	0.9600
C3—H3B	0.9600	C58—H58C	0.9600
C3—H3C	0.9600	C59—H59A	0.9600
C4—H4A	0.9600	C59—H59B	0.9600
C4—H4B	0.9600	C59—H59C	0.9600
C4—H4C	0.9600	C60—H60A	0.9600
C5—H5A	0.9600	C60—H60B	0.9600
C5—H5B	0.9600	C60—H60C	0.9600
C5—H5C	0.9600	C61—H61A	0.9600
C6—H6A	0.9600	C61—H61B	0.9600
C6—H6B	0.9600	C61—H61C	0.9600
C6—H6C	0.9600	C62—H62A	0.9600
C7—H7A	0.9600	C62—H62B	0.9600
C7—H7B	0.9600	C62—H62C	0.9600
C7—H7C	0.9600	C63—H63A	0.9600
C8—H8A	0.9600	C63—H63B	0.9600
C8—H8B	0.9600	C63—H63C	0.9600
C8—H8C	0.9600	C64—H64A	0.9600
C9—H9A	0.9600	C64—H64B	0.9600
C9—H9B	0.9600	C64—H64C	0.9600
C9—H9C	0.9600	C65—H65A	0.9600
C10—H10A	0.9600	C65—H65B	0.9600
C10—H10B	0.9600	C65—H65C	0.9600
C10—H10C	0.9600	C66—H66A	0.9600
C11—H11A	0.9600	C66—H66B	0.9600
C11—H11B	0.9600	C66—H66C	0.9600
C11—H11C	0.9600	C67—H67A	0.9600
C12—H12A	0.9600	C67—H67B	0.9600
C12—H12B	0.9600	C67—H67C	0.9600
C12—H12C	0.9600	C68—H68A	0.9600
C13—H13A	0.9600	C68—H68B	0.9600
C13—H13B	0.9600	C68—H68C	0.9600
C13—H13C	0.9600	C69—H69A	0.9600
C14—H14A	0.9600	C69—H69B	0.9600
C14—H14B	0.9600	C69—H69C	0.9600
C14—H14C	0.9600	C70—H70A	0.9600
C15—H15A	0.9600	C70—H70B	0.9600
C15—H15B	0.9600	C70—H70C	0.9600
C15—H15C	0.9600	C71—H71A	0.9600
C16—H16A	0.9600	C71—H71B	0.9600
C16—H16B	0.9600	C71—H71C	0.9600
C16—H16C	0.9600	C72—H72A	0.9600
C17—H17A	0.9600	C72—H72B	0.9600
C17—H17B	0.9600	C72—H72C	0.9600
C17—H17C	0.9600	Ce4—O34	2.350 (6)
C18—H18A	0.9600	Ce4—O32	2.360 (5)
C18—H18B	0.9600	Ce4—O33	2.365 (5)
C18—H18C	0.9600	Ce4—O31	2.398 (5)
C19—H19A	0.9600	Ce4—O40	2.583 (6)
C19—H19B	0.9600	Ce4—O38	2.606 (6)
C19—H19C	0.9600	Ce4—O35	2.609 (6)
C20—H20A	0.9600	Ce4—O37	2.620 (6)
C20—H20B	0.9600	Ce4—N55	3.025 (7)
C20—H20C	0.9600	Ce4—N56	3.028 (7)
C21—H21A	0.9600	P13—O31	1.488 (6)
C21—H21B	0.9600	P13—N44	1.633 (7)
C21—H21C	0.9600	P13—N45	1.634 (6)
C22—H22A	0.9600	P13—N43	1.635 (7)
C22—H22B	0.9600	P14—O32	1.499 (5)
C22—H22C	0.9600	P14—N48	1.612 (7)
C23—H23A	0.9600	P14—N47	1.631 (6)
C23—H23B	0.9600	P14—N46	1.668 (6)
C23—H23C	0.9600	P15—O33	1.501 (5)
C24—H24A	0.9600	P15—N49	1.623 (7)
C24—H24B	0.9600	P15—N50	1.625 (7)
C24—H24C	0.9600	P15—N51	1.634 (8)
Ce2—O12	2.368 (5)	P16—O34	1.504 (6)
Ce2—O13	2.372 (5)	P16—N52	1.617 (7)
Ce2—O14	2.380 (5)	P16—N53	1.626 (7)
Ce2—O11	2.411 (5)	P16—N54	1.633 (7)
Ce2—O20	2.588 (6)	O35—N55	1.309 (9)
Ce2—O15	2.596 (6)	O36—N55	1.228 (9)
Ce2—O18	2.610 (6)	O37—N55	1.257 (9)
Ce2—O17	2.610 (6)	O38—N56	1.294 (9)
Ce2—N28	3.018 (8)	O39—N56	1.213 (9)
Ce2—N27	3.019 (7)	O40—N56	1.275 (9)
P5—O11	1.487 (5)	N43—C73	1.464 (10)
P5—N17	1.624 (7)	N43—C74	1.466 (9)
P5—N15	1.642 (7)	N44—C75	1.453 (10)
P5—N16	1.644 (7)	N44—C76	1.469 (10)
P6—O12	1.497 (5)	N45—C77	1.468 (9)
P6—N19	1.623 (7)	N45—C78	1.473 (10)
P6—N20	1.630 (6)	N46—C80	1.469 (8)
P6—N18	1.645 (6)	N46—C79	1.473 (9)
P7—O13	1.488 (6)	N47—C81	1.432 (10)
P7—N21	1.617 (7)	N47—C82	1.457 (9)
P7—N23	1.635 (8)	N48—C84	1.461 (10)
P7—N22	1.637 (7)	N48—C83	1.479 (10)
P8—O14	1.483 (6)	N49—C86	1.459 (10)
P8—N24	1.633 (7)	N49—C85	1.476 (10)
P8—N25	1.635 (7)	N50—C88	1.465 (10)
P8—N26	1.640 (7)	N50—C87	1.483 (10)
O15—N27	1.268 (9)	N51—C90	1.436 (10)
O16—N27	1.212 (9)	N51—C89	1.468 (12)
O17—N27	1.279 (9)	N52—C92	1.449 (11)
O18—N28	1.266 (9)	N52—C91	1.495 (11)
O19—N28	1.242 (9)	N53—C94	1.457 (10)
O20—N28	1.260 (10)	N53—C93	1.461 (11)
N15—C25	1.454 (10)	N54—C95	1.447 (10)
N15—C26	1.465 (10)	N54—C96	1.470 (10)
N16—C28	1.459 (10)	C73—H73A	0.9600
N16—C27	1.467 (10)	C73—H73B	0.9600
N17—C29	1.446 (10)	C73—H73C	0.9600
N17—C30	1.481 (11)	C74—H74A	0.9600
N18—C31	1.448 (9)	C74—H74B	0.9600
N18—C32	1.470 (9)	C74—H74C	0.9600
N19—C33	1.438 (9)	C75—H75A	0.9600
N19—C34	1.465 (10)	C75—H75B	0.9600
N20—C35	1.451 (10)	C75—H75C	0.9600
N20—C36	1.476 (10)	C76—H76A	0.9600
N21—C37	1.449 (11)	C76—H76B	0.9600
N21—C38	1.462 (11)	C76—H76C	0.9600
N22—C40	1.427 (11)	C77—H77A	0.9600
N22—C39	1.457 (10)	C77—H77B	0.9600
N23—C41	1.465 (11)	C77—H77C	0.9600
N23—C42	1.467 (12)	C78—H78A	0.9600
N24—C43	1.448 (11)	C78—H78B	0.9600
N24—C44	1.476 (11)	C78—H78C	0.9600
N25—C45	1.467 (10)	C79—H79A	0.9600
N25—C46	1.473 (11)	C79—H79B	0.9600
N26—C48	1.441 (11)	C79—H79C	0.9600
N26—C47	1.462 (13)	C80—H80A	0.9600
C25—H25A	0.9600	C80—H80B	0.9600
C25—H25B	0.9600	C80—H80C	0.9600
C25—H25C	0.9600	C81—H81A	0.9600
C26—H26A	0.9600	C81—H81B	0.9600
C26—H26B	0.9600	C81—H81C	0.9600
C26—H26C	0.9600	C82—H82A	0.9600
C27—H27A	0.9600	C82—H82B	0.9600
C27—H27B	0.9600	C82—H82C	0.9600
C27—H27C	0.9600	C83—H83A	0.9600
C28—H28A	0.9600	C83—H83B	0.9600
C28—H28B	0.9600	C83—H83C	0.9600
C28—H28C	0.9600	C84—H84A	0.9600
C29—H29A	0.9600	C84—H84B	0.9600
C29—H29B	0.9600	C84—H84C	0.9600
C29—H29C	0.9600	C85—H85A	0.9600
C30—H30A	0.9600	C85—H85B	0.9600
C30—H30B	0.9600	C85—H85C	0.9600
C30—H30C	0.9600	C86—H86A	0.9600
C31—H31A	0.9600	C86—H86B	0.9600
C31—H31B	0.9600	C86—H86C	0.9600
C31—H31C	0.9600	C87—H87A	0.9600
C32—H32A	0.9600	C87—H87B	0.9600
C32—H32B	0.9600	C87—H87C	0.9600
C32—H32C	0.9600	C88—H88A	0.9600
C33—H33A	0.9600	C88—H88B	0.9600
C33—H33B	0.9600	C88—H88C	0.9600
C33—H33C	0.9600	C89—H89A	0.9600
C34—H34A	0.9600	C89—H89B	0.9600
C34—H34B	0.9600	C89—H89C	0.9600
C34—H34C	0.9600	C90—H90A	0.9600
C35—H35A	0.9600	C90—H90B	0.9600
C35—H35B	0.9600	C90—H90C	0.9600
C35—H35C	0.9600	C91—H91A	0.9600
C36—H36A	0.9600	C91—H91B	0.9600
C36—H36B	0.9600	C91—H91C	0.9600
C36—H36C	0.9600	C92—H92A	0.9600
C37—H37A	0.9600	C92—H92B	0.9600
C37—H37B	0.9600	C92—H92C	0.9600
C37—H37C	0.9600	C93—H93A	0.9600
C38—H38A	0.9600	C93—H93B	0.9600
C38—H38B	0.9600	C93—H93C	0.9600
C38—H38C	0.9600	C94—H94A	0.9600
C39—H39A	0.9600	C94—H94B	0.9600
C39—H39B	0.9600	C94—H94C	0.9600
C39—H39C	0.9600	C95—H95A	0.9600
C40—H40A	0.9600	C95—H95B	0.9600
C40—H40B	0.9600	C95—H95C	0.9600
C40—H40C	0.9600	C96—H96A	0.9600
C41—H41A	0.9600	C96—H96B	0.9600
C41—H41B	0.9600	C96—H96C	0.9600
C41—H41C	0.9600		

Symmetry codes: (i) *x*, *y*, *z*−1; (ii) *x*, *y*, *z*+1.
